# Depression of LncRNA DANCR alleviates tubular injury in diabetic nephropathy by regulating KLF5 through sponge miR-214-5p

**DOI:** 10.1186/s12882-024-03562-6

**Published:** 2024-04-12

**Authors:** Yongling Kuang, Juan Yang, Meimei Sun, Tingting Rui, Zhenhua Yang, Meihua Shi

**Affiliations:** Department of Nephrology, Gongli Hospital of Shanghai Pudong New Area, No. 219 Miaopu Road, Pudong New Area, 200135 Shanghai, China

**Keywords:** Diabetic nephropathy, DANCR, miR-214-5p, Diagnostic, KLF5

## Abstract

**Objective:**

Diabetic nephropathy (DN) manifests a critical aspect in the form of renal tubular injury. The current research aimed to determine the function and mechanism of long non-coding ribonucleic acid (LncRNA) differentiation antagonising non-protein coding RNA (DANCR), with a focus on its impact on renal tubular injury.

**Methods:**

Quantitative reverse transcription polymerase chain reaction was employed to analyze the RNA levels of DANCR in the serum of patients with DN or human proximal tubular epithelial cells (human kidney 2 [HK2]). The diagnostic significance of DANCR was assessed using a receiver operating characteristic curve. A DN model was established by inducing HK-2 cells with high glucose (HG). Cell proliferation, apoptosis, and the levels of inflammatory factors, reactive oxygen species (ROS), and malondialdehyde (MDA) were detected using the Cell Counting Kit − 8, flow cytometry, and enzyme-linked immunosorbent assay. The interaction between microRNA (miR)-214-5p and DANCR or Krüppel-like factor 5 (KLF5) was investigated using RNA immunoprecipitation and dual-luciferase reporter assays.

**Results:**

Elevated levels of DANCR were observed in the serum of patients with DN and HG-inducted HK-2 cells (*P* < 0.05). DANCR levels effectively identified patients with DN from patients with type 2 diabetes mellitus. Silencing of DANCR protected against HG-induced tubular injury by restoring cell proliferation, inhibiting apoptosis, and reducing the secretion of inflammatory factors and oxidative stress production (*P* < 0.05). DANCR functions as a sponge for miR-214-5p, and the mitigation of DANCR silencing on HG-induced renal tubular injury was partially attenuated with reduced miR-214-5p (*P* < 0.05). Additionally, KLF5 was identified as the target of miR-214-5p.

**Conclusion:**

DANCR was identified as diagnostic potential for DN and the alleviation of renal tubular injury via the miR-214-5p/KLF5 axis, following DANCR silencing, introduces a novel perspective and approach to mitigating DN.

**Supplementary Information:**

The online version contains supplementary material available at 10.1186/s12882-024-03562-6.

## Introduction

The global incidence of kidney disease-related deaths is on a concerning upward trajectory [1]. Diabetic nephropathy (DN) also named diabetic kidney disease (DKD), stands as the most prevalent microvascular complication associated with diabetes and constitutes a significant contributor to end-stage renal failure [2]. With an anticipated worldwide diabetic population of 783 million in 2024 [3], approximately 30–40% are expected to develop DN [4, 5]. DN, marked by early symptoms and rapid progression, poses a substantial risk of uraemia, a critical factor in diabetes mellitus (DM)-related disability or mortality [6]. The prevailing current clinical diagnoses of DN predominantly rely on persistent albuminuria and diminished estimated glomerular filtration rate (eGFR) [7]. However, these indicators possess limited diagnostic value and fail to identify DM patients with microvascular complications before the onset of renal injury [8]. Consequently, DN is usually confirmed during autopsy [6]. Therefore, the imperative for early identification and timely intervention in the progression of DN necessitates ongoing research and exploration of novel, feasible biomarkers, and therapeutic targets.

Long non-coding ribonucleic acids (RNAs) (LncRNAs) are RNA molecules devoid of protein-coding ability and play a significant role in the progression of various diseases by functioning as competing endogenous RNAs (ceRNAs) for microRNAs (miRNAs/miR) [9]. For example, LncRNAs such as MSC antisense RNA 1 [10], DLX6 antisense RNA 1 [11], and nuclear paraspeckle assembly transcript 1 (NEAT1) [12] operate as ceRNAs in DN progression. One noteworthy lncRNA in this context is differentiation antagonising non-protein coding RNA (DANCR), also known as AGU2 or small nucleolar RNA host gene (SNHG) 13, located on human chromosome 4q12. DANCR has been previously studied in hepatocellular carcinoma [13], breast cancer [14], and prostate cancer [15], and demonstrated essential roles in vascular disease. Recent research has underscored DANCR’s significance in cerebral microvascular endothelial injury after ischemic stroke [16], and angiogenesis in ovarian cancer [17]. Elevated DANCR levels have been observed in patients with atherosclerosis, where it serves as a predictive biomarker [18]. DANCR inhibition typically alleviates oxygen-glucose deprivation-induced myocardial injury [19]. Inflammation exacerbates DN progression, and DANCR dysregulation has been reported in osteoarthritis [20], colitis [21], and severe acute respiratory syndrome coronavirus infection [22]. Furthermore, DANCR is involved in regulating immune tolerance in mouse kidney transplantation via mesenchymal stem cell-derived exosomes [23]. Notably, Gao et al. identified differentially expressed LncRNAs in DN through microarray database analysis, including DANCR, which was associated with DN pathogenesis [24]. However, the precise mechanism of DANCR action in DN remains unclear.

Consequently, it was hypothesized that DANCR might exacerbate DN progression. The clinical value and potential mechanism of action of DNACR in DN were explored to verify this hypothesis, providing a new theoretical basis for its diagnosis and treatment.

## Materials and methods

### Ethics statement

All participants completed an informed consent form prior to participation in the present research. Ethical approval for this study was provided by the Ethics Committee of the Gongli Hospital of Shanghai Pudong New Area (approval number: 2019-011). Also, the study was conducted in compliance with the Declaration of Helsinki.

### Participants and data collection

Patients with type 2 DM (T2DM) admitted to Gongli Hospital of Shanghai Pudong New Area Hospital between January 2019 and December 2020 were included in this study. T2DM diagnosis adhered to the American Diabetes Association criteria [25]: (a) fasting blood glucose (FBG) ≥ 7.0 mmol/L; (b) blood glucose 2 h after glucose load ≥ 11.1 mmol/L or glycated haemoglobin A1c (HbA1c) ≥ 6.5%. Among them, patients with T2DM were divided into two groups: those with DN (*n* = 68) and those without DN (DM, *n* = 55). DN inclusion criteria were as follows: (a) history of T2DM; (b) persistent albuminuria (≥ 30 mg/24 h) or a random albuminuria to creatinine ratio of ≥ 30 mg/g or eGFR < 60 mL/min/1.73 m^2^; (c) age 18–70 years. The exclusion criteria were as follows: (a) type I DM or other diabetic complications; (b) concurrent chronic kidney diseases such as glomerulonephritis; (c) autoimmune diseases, malignant tumors, and haematologic diseases; (d) presence of cardiovascular diseases (myocardial infarction, unstable angina); (e) use application of glucocorticoids, or immunosuppressive drugs within the last 6 months. Additionally, 50 healthy volunteers (HVs) were included as controls, matched for age and gender with the patient groups. Individuals with DM, cardiovascular disease, renal disease, autoimmune disease, and those using antibiotics or corticosteroids were excluded. Demographic characteristics and clinical baseline information of the participants are presented in Table 1.

**Table 1:** Comparison of the baseline data of the subject

Variables	HVs (*n* = 50)	DM (*n* = 55)	DN (*n* = 68)	*P* value
Age (year)	51.92 ± 12.94	55.56 ± 10.59	53.88 ± 12.50	0.305
Gender, male (%)	29 (58.00)	30 (54.55)	41 (60.29)	0.813
BMI (kg/m^2^)	26.86 ± 3.44	25.64 ± 4.23	25.92 ± 4.68	0.302
Smoking (%)	33 (66.00)	29 (52.73)	38 (55.89)	0.357
Alcohol (%)	28 (56.00)	33 (60.00)	38 (55.89)	0.881
Hypertension				
SBP (mmHg)	121.08 ± 20.74	126.80 ± 12.42	121.38 ± 16.65	0.132
DBP (mmHg)	75.49 ± 7.74	77.48 ± 7.62	78.81 ± 7.20	0.062
Lipid profiles				
TC (mg/dL)	180.81 ± 21.76	170.31 ± 46.25	181.96 ± 49.74	0.271
LDL cheolesterol (mg/dL)	109.72 ± 27.59	110.37 ± 18.47	112.51 ± 16.41	0.838
HDL cheolesterol (mg/dL)	50.92 ± 8.26	48.76 ± 8.45	48.90 ± 8.14	0.323
Glucose metabolism				
FBG (mmol/L)	5.21 ± 1.41	8.76 ± 0.81	6.70 ± 0.77	< 0.001
HbA1c (%)	5.76 ± 0.87	9.95 ± 2.34	9.67 ± 2.28	< 0.001
Laboratory values				
Hemoglobin (g/dL)	13.30 ± 3.49	13.94 ± 1.62	13.97 ± 1.91	0.489
BUN (mg/dL)	11.34 ± 2.67	15.08 ± 3.84	17.76 ± 6.88	< 0.001
Creatinine (mg/dL)	62.75 ± 3.67	62.87 ± 4.07	378.13 ± 60.67	< 0.001
eGFR (mL/min/1.73m^2^)	85.85 ± 13.45	86.09 ± 14.14	56.19 ± 16.04	< 0.001
Albuminuria (mg/24 h)	2.70 ± 0.74	7.45 ± 2.42	580.88 ± 333.04	< 0.001
hs-CRP (g/L)	0.58 ± 0.15	5.17 ± 1.69	130.58 ± 55.38	< 0.001

Note: HVs, healthy individuals; DM, T2DM patients; DN, Diabetic nephropathy patients

BMI: body mass index; SBP: systolic blood pressure; DBP: Diastolic blood pressure; TC: total cholesterol; LDL: Low-density lipoprotein; HDL: high density lipoprotein; FBG: fasting blood glucose; HbA1c: glycated hemoglobin A1c; BUN: blood urea nitrogen; hs-CRP: high-sensitivity C-reactive protein; eGFR: estimated glomerular filtration rate

### Specimen collection and biochemical

Venous blood was obtained from the upper extremities of the participants after 8 h of fasting. A portion of the collected blood was stored in anticoagulation tubes for HbA1c levels. The remaining blood was allowed to stand at room temperature and then centrifuged at 3000 g for 10 min to obtain the upper serum. Using a biochemistry analyser (Instrumentation Laboratory, USA), various indicators such as FBG, total cholesterol (TC), and triglyceride (TG)were measured. Additionally, a part of the serum was stored at -80℃ for the analysis of DANCR and miRNA messenger RNA levels. Urine samples (10 mL) were collected and centrifuged at 400 g for the determination of urine albumin and other levels.

### Cell culture and high glucose (HG) treatment

Human proximal tubular epithelial cells (human kidney 2 [HK-2]) were procured from the BeNa Culture Collection (cat: BNCC339833, China). These cells were cultured in Dulbecco’s Modified Eagle Medium (DMEM, cat: C11995500, Invitrogen, USA) supplemented with 10% fetal bovine serum (cat: 16-000-044, Invitrogen, USA), and 1% penicillin/streptomycin (cat: 1,510,122, Invitrogen, USA). The cells were maintained in a humidified incubator with 5% carbon dioxide at 37℃. HK-2 cells were treated with HG (30 mM glucose, cat: G-8769, Sigma-Aldrich, USA) for 12 h, 24 h, and 36 h. The control group was exposed to normal glucose (NG, 5.5 mM glucose + 24.5 mM mannitol, cat: M-1902, Sigma-Aldrich, USA).

### Cell transfection

Transfection was initiated upon achieving a cell fusion rate reached of 70%. Small interfering (si) RNA targeting DANCR (si-DANCR) and its negative control (si-NC) or, as well as the miR-214-5p mimic, miR-214-5p inhibitor, and their negative control (mimic NC and inhibitor NC, obtained from GenePharma, China), were combined with the transfection reagent Lipofectamine 3000 (cat: 1,662,152, Invitrogen, USA) in DMEM. The mixture was then allowed to incubate for 20 min at room temperature. Subsequently, the prepared mixture was introduced into the cells, and the incubation continued for 6 h before replacing the DMEM.

### Quantitative reverse transcription polymerase chain reaction (PCR) (RT-qPCR)

The TRIzol LS Reagent (cat: 15,596,026, Invitrogen, USA) was used for pre-incubation, followed by purification using the miRNeasy Serum/Plasma Kit (cat: Q217184, Qiagen, Germany) to extract total RNA from 500 μL of serum. Meanwhile, for the cells, the miRNeasy Mini Kit (cat: Q217004, Qiagen, Germany) was used to purify total RNA from the cells. The concentration and purity of the extracted RNA were determined using the NanoDrop2000 micro ultraviolet spectrophotometer (NanoDrop Technologies, USA). The reverse transcription of RNA into complementary deoxyribonucleic acid (cDNA) was performed using M-MLV Reverse Transcriptase (cat: M170B, Promega, USA) or the miRcute Plus miRNA First-Strand cDNA Kit (cat: KR211, TIANGEN Biotech, China). Amplification reactions were performed by combining SuperReal PreMix Plus (SYBR Green) (cat: FP205, TIANGEN Biotech, China) or the miRcute Plus miRNA qPCR Kit (SYBR Green) (cat: KR411, TIANGEN Biotech, China) with primers using cDNA as a template. Where β-actin and U6 were normalized separately, and the quantification was performed using the 2^−ΔΔCt^ method.

### Western blot assay

Transfected and HG-induced HK-2 cells were spiked with RIPA lysis buffer (cat: P0013B, Beyotime, China), and the supernatant was collected after centrifugation to extract total protein. The protein concentration was analyzed with a BCA protein quantitation assay kit (cat: P0012, Beyotime, China), followed by denaturation by mixing the protein with 10% alkyl sodium sulfate buffer in a certain ratio and boiling for 5 min in a 95℃-water bath. Electrophoretic separation was performed using SDS-PAGE gels and transferred to 0.22 μm PVDF membranes (cat: ISEQ00010, Millipore, USA). Then, the membranes were blocked for 2 h at room temperature in 5% bovine serum albumin (BSA)(cat: A9647, Millipore, USA), followed by incubation with primary antibodies (KFL5 antibody, cat: 668,501-Ig, Proteintech, China; β-actin antibody, cat: 81115-1-RR, Proteintech, China) at 1:1000 dilutions overnight at 4℃. After washing with 0.05% tris buffered saline/Tween (TBST), the membrane was incubated with enzyme-labeled secondary antibody (Proteintech, China, 1:5000 dilution) for 2 h. Finally, proteins were displayed using the enhanced chemiluminescent luminescence (ECL) kit (cat: NEL103001EA, Perkin Elmer, USA) and protein band images were analyzed using the Bio-Rad ChemiDOC XRS system (Bio-Rad Corporation, USA). The original gel chart has been presented in the supplementary material.

### Cell proliferation assay

Transfected and HG-induced HK-2 cells were converted into cell suspensions, and 1 × 10^4^ cells were inoculated into 96-well plates for incubation. Before the assessment, 10 μL of cell count kit-8 solution (cat: KB491, Dojindo Laboratories, Japan) was added to the cells, and the incubation process was continued for 1 h. The cell proliferation rate was determined by measuring the optical density value at 450 nm.

### Cell apoptosis assay

Cells were inoculated into six-well plates, and 24 h later, HG was introduced, followed by the transfection of plasmids. After 72 h, the cells were collected and washed with pre-cooled phosphate-buffered saline (PBS) and the binding buffer was added. Annexin V and propidium iodide from an Annexin V-fluorescein isothiocyanate (FITC)/propidium iodide cell apoptosis kit (cat: 556,547, BD, USA) kit was then stained in a darkroom for 10 min. The quantification of apoptotic cells was performed using flow cytometry (BD-Biosciences, USA).

### Enzyme-linked immunosorbent assay (ELISA)

Serum from the participants and the supernatant from HG-inducted and transfected HK-2 cells were collected. The expression levels of interleukin (IL) -1β (cat: E-EL-H0149c, Elabscience Biotech, China), IL-6 (cat: E-EL-H6156, Elabscience Biotech, China), and tumor necrosis factor α (TNF-α, Cat: E-EL-H0109c, Elabscience Biotech, China) were measured according to the manufacturer’s instructions.

### Reactive oxygen species (ROS) and malondialdehyde (MDA) measurement

The fluorescent probe 2’,7’-dichlorofluorescein diacetate (DCFH-DA) kit (cat: E004-1-1, Nanjing Jiancheng Bioengineering Institute, China) was used to detect ROS level according to the manufacturer’s instructions. Transfected and HG-induced HK-2 cells were incubated with 40 μL of DCFH-DA buffer for 30 min at 37℃. Following the incubation, the cells were washed, and the fluorescence intensity was quantified at wavelengths of 488 nm and 525 nm to assess ROS levels.

The MDA levels were analyzed using the thiobarbituric acid (TBA) method by an MDA kit (cat: A003-1-1, Nanjing Jiancheng Bioengineering Institute, China) according to the manufacturer’s recommendations. In short, MDA powder was added to 32 mL of hot distilled water at 90–100℃ and fully dissolved. After cooling, 30 mL of glacial acetic acid was added and mixed. A 2:1 dilution was made with 50% glacial acetic acid and frozen in a 4℃-refrigerator protected from light as a working solution. The cells of Transfected and HG-induced HK-2 cells were crushed, shaken well, and then taken and added to the mixture of reagents containing the working solution. 95℃ water bath for 40 min, removed and cooled with running water, 3500  rpm, centrifuged for 10 min, then the supernatant was taken and detected the absorbance value at 532 nm.

### Subcellular fractional location

The PARIS kit (cat: AM1921, Thermo Scientific, USA) was employed to explore the localization of DANCR on the HK-2 cells. In brief, cells were washed with PBS, centrifuged at 1000 rpm for 5 min, and harvested by removing PBS. Pre-cooled cell lysate was added, and the mixture was incubated for 1 min. The supernatant, obtained by centrifugation at 1200 rpm for 5 min, was used for cytoplasmic fragment RNA assay. The remaining precipitate was treated with the NER reagent from the kit, incubated for 10 min, and then used for collecting nuclear RNA. U6 and β-actin served as positive controls for the nucleus and cytoplasm. RT-qPCR was designed to assess DANCR levels in the cytoplasm and nucleus fractions.

### Dual luciferase reporter (DLR) assay

The promoter sequences of DANCR or Krüppel-like factor 5 (KLF5) were cloned into pmirGLO Vectors to construct a wild-type (WT) DANCR or KLF5 reporter plasmid (DANCR-WT or KLF5-WT). Additionally, the binding site was mutated to construct mutant (Mut) DANCR or KLF5 reporter plasmid (DANCR-Mut or KLF5-Mut). These recombinant plasmids were co-transfected with miR-214-5p mimic or miR-214-5p inhibitor using Lipofectamine 3000 (Invitrogen, USA). HK-2 cells for 48 h. Subsequently, luciferase activity was evaluated using a commercial DLR kit (cat: E1960, Promega, USA).

### RNA immunoprecipitation (RIP) assay

The RIP assay was performed to analyse the interaction between miR-214-5p, DANCR, and KLF5 by the RIP Assay Kit (cat: 17–704, Millipore, USA). HK-2 cells transfected with miR-214-5p mimic or mimic NC were lysed in RIP buffer and incubated overnight at 4℃ with magnetic beads coupled with human Ago2 or immunoglobulin G (IgG) antibodies. After the purification of co-precipitated RNA, RT-qPCR was employed to quantify the enrichment levels of DANCR and KLF5.

### Statistical analysis

The experimental data from three biological replicates are presented as the mean ± standard deviation. Statistical analysis and figure generation were performed using GraphPad Prism 7.0 and SPSS 23.0. Student’s t-test was employed to assess the differences between the two groups. Analysis of variance and Turkey’s analysis were used to compare the differences between multiple groups. Receiver operating characteristic (ROC) curve analysis was conducted for diagnostic prospect assessment. Pearson’s correlation coefficient was calculated for correlation detection. Statistical significance was set at *P* < 0.05.

## Results

### Demographic and clinical baseline characteristics

The demographic characteristics of the 68 patients with DN, 55 patients with DM, and 50 HVs enrolled in this study were initially examined. As presented in Table 1, there were no significant differences in age, gender, body mass index (BMI), smoking, and alcohol consumption (*P* > 0.05). Clinical baseline characteristics, including systolic blood pressure, diastolic blood pressure, lipid profile, and haemoglobin, did not show statistically significant differences among the three groups (*P* > 0.05). However, significant differences were observed in FBG, HbA1c, blood urea nitrogen, eGFR, albuminuria, and creatinine (*P* < 0.05).

### LncRNA DANCR was enhanced in patients with DN and has diagnostic potential

**Fig. 1:**
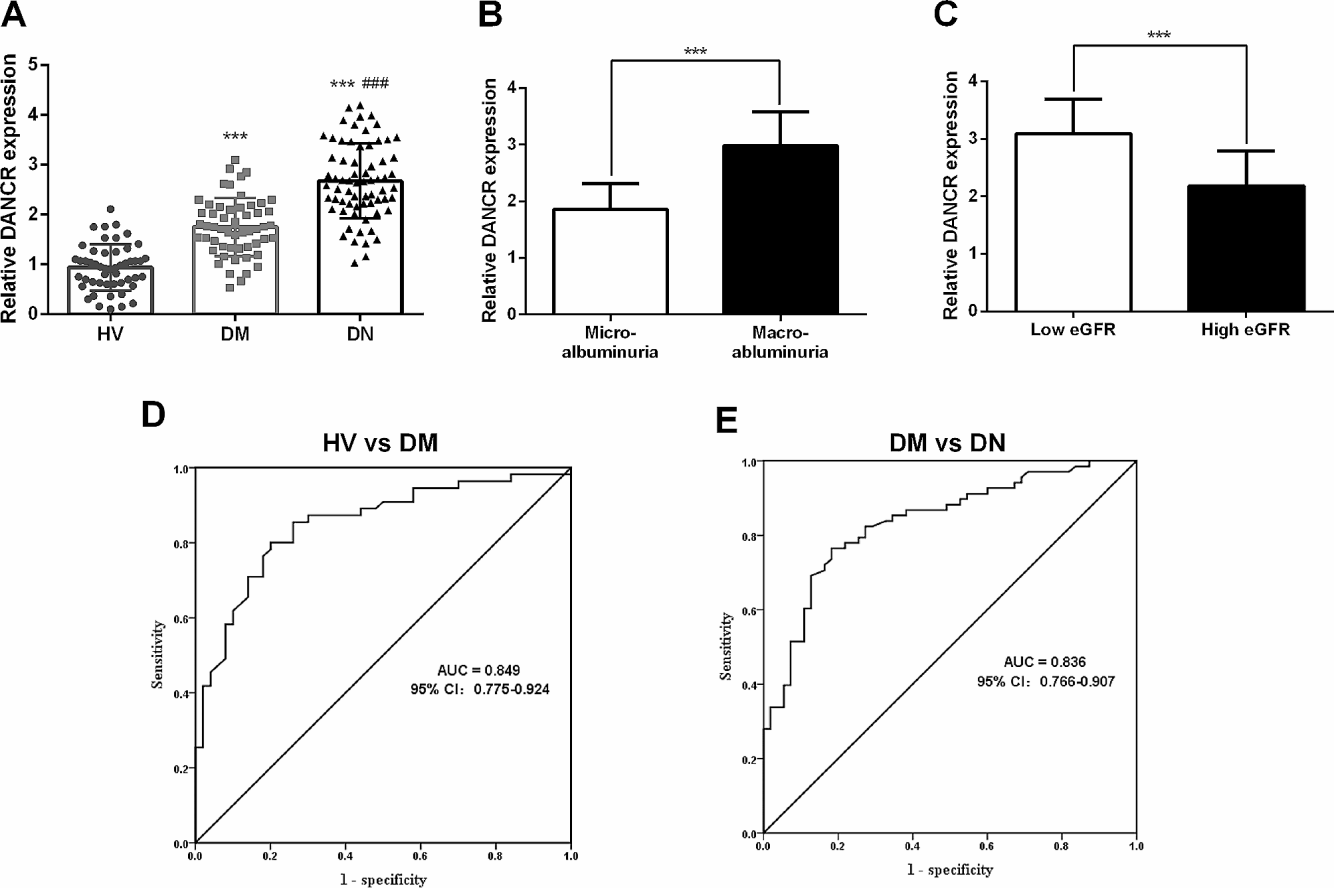
Expression and diagnostic potential of long non-coding ribonucleic acid (RNA) differentiation antagonizing non-protein coding RNA (DANCR) in diabetic nephropathy (DN). **A**. Serum DANCR was typically elevated in patients with diabetes mellitus without DN (DM) and DN, and more significantly in DN. **B**. Quantitative reverse transcription polymerase chain reaction was conducted to explore the DANCR levels in DN patients with micro-albuminuria or macro-albuminuria. **C**. Detected serum DANCR expression in high and low estimated glomerular filtration rate (eGFR) groups based on the mean eGFR values. The receiver operating characteristic curve was applied to analyze the diagnostic value of serum DANCR in differentiating DM from healthy volunteers (HVs) (**D**) and DN from DM (**E**). *** *P* < 0.001 vs. HV, micro-albuminuria, or low eGFR groups; ### *P* < 0.001 vs. DM group

As indicated by RT-qPCR, serum DANCR levels were consistently elevated in patients with DM and DN compared to HVs (*P* < 0.01). Moreover, patients with DN exhibited significantly higher DANCR levels compared to patients with DM (*P* < 0.01, Fig. 1A). Compared to micro-albuminuria, DN patients with macro-albuminuria demonstrated elevated DANCR levels compared to those with microproteinuria (*P* < 0.05, Fig. 1B). Upon categorizing patients with DN into low eGFR groups based on the mean eGFR value, statistically higher DANCR levels were observed in patients with low eGFR (*P* < 0.05, Fig. 1C). Given that eGFR and albuminuria are well-established biomarkers for DN [26], the diagnostic potential of DANCR in DN was subsequently evaluated. ROC analysis confirmed that DANCR exhibited a sensitivity and specificity of 80% each in distinguishing patients with DM from HVs, with an area under the curve (AUC) of 0.849 (95% confidence interval [CI]: 0.775–0.924, Fig. 1D). Similarly, DANCR demonstrated a sensitivity and specificity of 76.5% and 80%, respectively, in distinguishing patients with DN from those with DM, resulting in an AUC of 0.836 (95%CI: 0.766–0.927, Fig. 1E), indicating a robust diagnostic potential.

### Silencing DANCR ameliorates HG-induced renal tubular epithelial cell injury

**Fig. 2:**
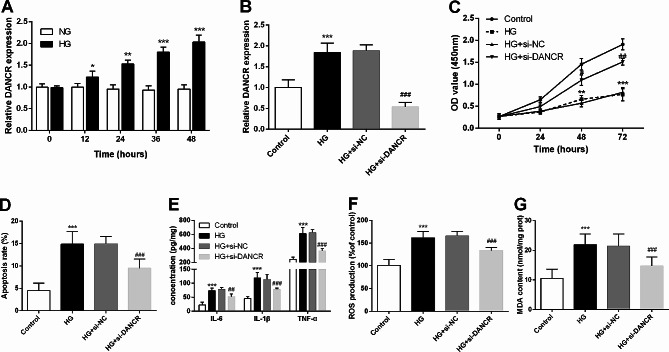
Silencing of differentiation antagonizing non-protein coding ribonucleic acid (DANCR) alleviates high glucose (HG)-induced renal tubular epithelial cell injury. **A**. The DANCR levels gradually increased with prolonged HG induction. **B**. In HG-induced human kidney 2 (HK-2) transfection with small interfering (si)-DANCR, a quantitative reverse transcription polymerase chain reaction was performed to investigate DANCR levels. HG-induced and si-DANCR-transfected HK-2 cell proliferation and apoptosis were detected using the Cell Counting Kit-8 (**C**) and flow cytometry (**D**). Enzyme-linked immunosorbent assay kits and commercial kits were used to evaluate the secretion of inflammatory factors (**E**), reactive oxygen species production (**F**), and malondialdehyde content (**G**). *** *P* < 0.001 vs. control; ### *P* < 0.001 vs. HG + si-negative control

An in vitro DN tubular injury model was established in HG-induced HK-2 cells to evaluate the potential contribution of DANCR in DN. As illustrated in Fig. 2A, the levels of DANCR gradually increased with prolonged HG induction (*P* < 0.001). Subsequently, HG induction for 36 h was chosen for further investigation. Compared with the HG + si-NC group, si-DANCR significantly inhibited the HG-induced elevation of DANCR levels (*P* < 0.001, Fig. 2B). Furthermore, HG induction led to impaired proliferation and increased apoptosis of HK-2 cells; however, these effects were significantly reversed by DANCR interference (*P* < 0.001, Fig. 2C-D). Additionally, HG induction enhanced the over-secretion of IL-6, TNF-α, and IL-1β, along with elevated ROS production and MDA content. However, these enhancements were substantially attenuated by si-DANCR (*P* < 0.05, Fig. 2E-G).

### DANCR functioned as a mir-214-5p sponge in DN

**Fig. 3:**
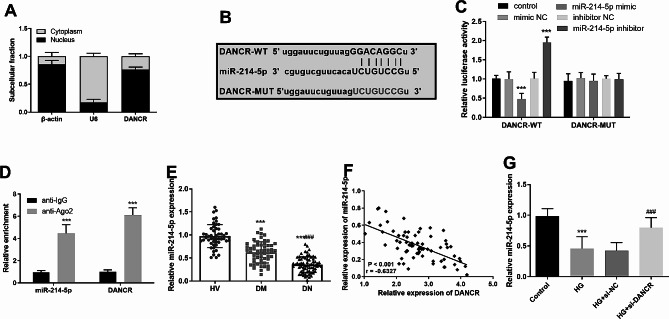
Differentiation antagonizing non-protein coding ribonucleic acid (RNA) (DANCR) functioned as a microRNA (miR)-214-5p sponge in diabetic nephropathy (DN). **A**. Subcellular localization analysis of DANCR in human kidney 2 cells. **B**. Bioinformatics found potential binding sites between DANCR and miR-214-5p. The specificity target between DANCR and miR-214-5p was explored using dual luciferase reporter assays (**C**) and RNA immunoprecipitation assay (**D**). **E**. Quantitative reverse transcription polymerase chain reaction was used to identify the expression of serum miR-214-5p in the participants. **F**. Correlation analysis of DANCR levels and miR-214-5p in patients with DN. **G**. Expression of miR-214-5p in high glucose (HG)-induced and DANCR-suppressed HK-2 cells. *** *P* < 0.001 vs. health volunteer group or control; ### *P* < 0.001 vs. diabetes mellitus group or HG + mall interfering-negative control

LncRNAs might serve as ceRNAs and might serve down-regulated gene expression by interacting with miRNAs. The subcellular localization of DANCR was first analyzed to elucidate the potential mechanisms of DANCR in DN. As illustrated in Fig. 3A, DANCR predominantly localizes in the cytoplasm. Bioinformatics analysis identified potential binding sites of DANCR to miR-214-5p (Fig. 3B). Additionally, the miR-214-5p mimic decreased the luciferase activity of DANCR-WT, while the miR-214-5p inhibitor increased its luciferase activity (*P* < 0.05). Importantly, neither of them affected the luciferase activity of DANCR-MUT (*P* > 0.05, Fig. 3C). In the RIP assay, DANCR and miR-214-5p were significantly enriched on Ago complexes compared to controls with IgG (*P* < 0.05, Fig. 3D). Moreover, miR-214-5p was markedly downregulated in patients with DN compared to HVs and patients with DM (*P* < 0.05, Fig. 3E). Additionally, miR-214-5p levels were negatively associated with DANCR in patients with DN (*r* = − 0.6327, *P* < 0.05, Fig. 3F). Finally, the levels of miR-214-5p were upregulated when DANCR was reduced in HG-induced HK-2 cells (*P* < 0.05, Fig. 3G).

### DANCR promotes renal tubular injury by suppressing miR-214-5p

**Fig. 4:**
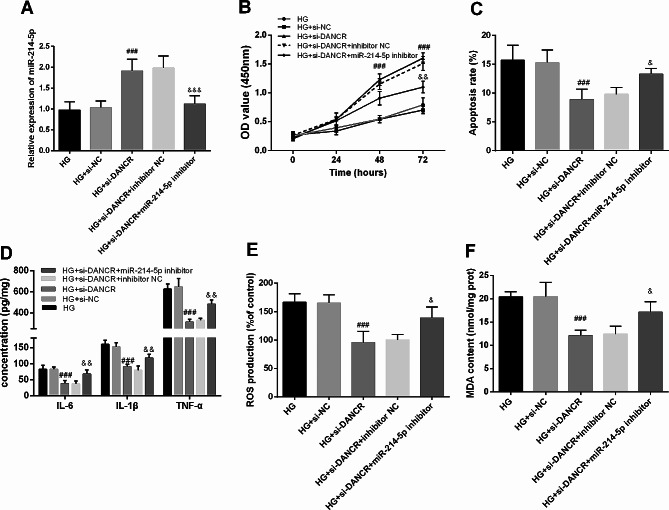
Differentiation antagonizing non-protein coding ribonucleic acid (RNA) (DANCR) promotes renal tubular injury by suppressing microRNA (miR)-214-5p. **A**. The level of miR-214-5p was detected after transfection with a miR-214-5p inhibitor in high glucose (HG)-induced and knocked-down DANCR in human kidney 2 (HK-2) cells. Cell Counting Kit-8 (**B**) and flow cytometry (**C**) were conducted to explore the proliferation and apoptosis of HK-2 cells after miR-214-5p suppression. Inflammatory factor secretion (**D**), reactive oxygen species production (**E**), and malondialdehyde components (**F**) were examined in HK-2 cells that inhibited miR-214-5p. ### *P* < 0.001 vs. HG + small interfering-negative control; & *P* < 0.05, && *P* < 0.01, &&& *P* < 0.001

To further investigate whether the impact of DANCR on renal tubular injury in DN is dependent on miR-214-5p, HG-induced HK-2 cells with DANCR knockdown were transfected with the miR-214-5p inhibitor. The results indicated that the upregulation of miR-214-5p by si-DANCR was significantly diminished in HG-induced HK-2 cells (*P* < 0.05, Fig. 4A). Additionally, si-DANCR significantly restored cell proliferation and inhibited apoptosis compared with the HG group (*P* < 0.05). However, reducing miR-214-5p reversed this effect (*P* < 0.05, Fig. 4B-C). The silencing miR-214-5p partially counteracted the decrease in HG-induced inflammatory secretion, superoxide dismutase production, and MDA content in HK-2 cells caused by si-DANCR (*P* < 0.05, Fig. 4D-F).

### DANCR modulates KLF5 expression by competitively binding to miR-214-5p

**Fig. 5:**
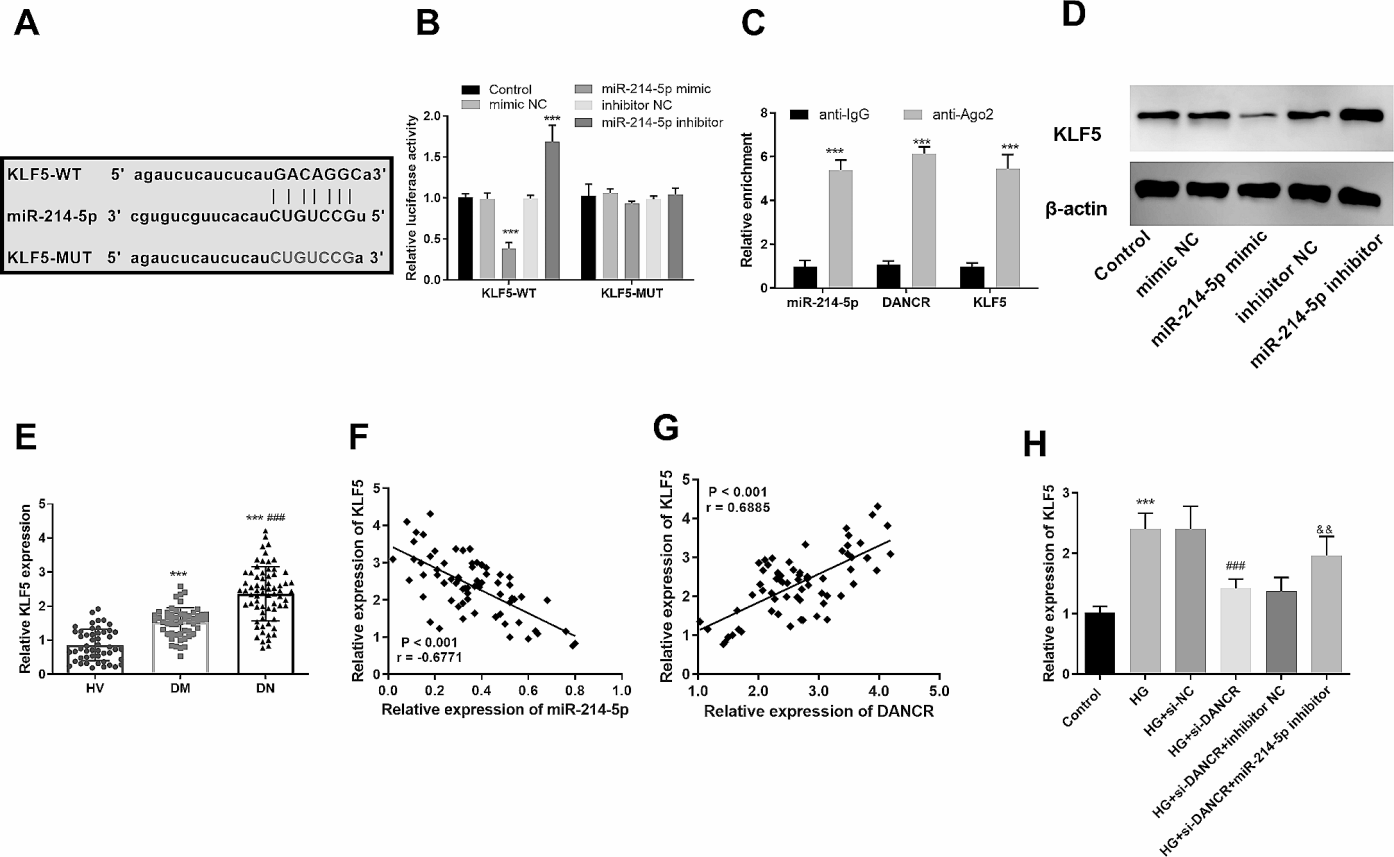
Differentiation antagonizing non-protein coding ribonucleic acid (RNA) (DANCR) modulates Krüppel-like factor 5 (KLF5) expression by competitively binding to microRNA (miR)-214-5p. **A**. Potential binding sites of miR-214-5p to KLF5. **B**. Dual luciferase reporter assay was adopted to examine the effect of miR-214-5p levels on the luciferase activity of KLF-5- wild type and KLF5-mutant. **C**. RNA immunoprecipitation assay was performed to analyze the enrichment between miR-214-5p, DANCR, and KLF5. **D**. Effect of miR-214-5p levels on KLF5 protein levels detected by Western blot. **E**. The KLF5 expression in the serum of the participants was analyzed. Correlation analysis of serum KLF5 levels with miR-214-5p (**F**) and DANCR (**G**) levels in patients with diabetic nephropathy, respectively. **H**. KLF5 levels after DANCR knockdown and inhibition of miR-214-5p in high glucose (HG)-induced HK-2 cells. *** *P* < 0.001 vs. healthy volunteer group or control; ### *P* < 0.001 vs. HG + small interfering negative control; & *P* < 0.05, && *P* < 0.01, &&& *P* < 0.001

To identify the target of miR-214-5p, bioinformatics analysis revealed potential binding sites for KLF5 (Fig. 5A). DLR assay that the miR-214-5p mimic or inhibitor significantly suppressed or enhanced the luciferase activity of KLF5-WT, with no significant impact on the luciferase activity of KLF5-MUT (*P* < 0.05, Fig. 5B). Additionally, the use of anti-Ago antibody increased the enrichment levels of miR-214-5p, DANCR, and KLF5 compared to the control anti-IgG group (*P* < 0.05, Fig. 5C). Additionally, compared to the control group, the protein expression of KLF5 was significantly inhibited by miR-214-5p mimic but increased by miR-214-5p inhibitor (*P* < 0.05, Fig. 5D). More importantly, KLF5 levels were significantly higher in patients with DN compared to HVs and patients with DM (*P* < 0.05, Fig. 5E). KLF5 levels were negatively correlated with miR-214-5p in patients with DN (*r* = -0.6771, *P* < 0.05, Fig. 5F), and positivity correlated with the DANCR levels (*r* = 0.6885, *P* < 0.05, Fig. 5G). Moreover, in HG-induced HK-2 cells, the inhibition of DANCR significantly reduced KLF5 levels, while suppression of miR-214-5p significantly rescued KLF5 levels (*P* < 0.05, Fig. 5H).

## Discussion

In DN, damage to renal blood vessels, tubules, and glomeruli leads to impaired renal function. While prior studies have primarily considered DN as a glomerular disorder, it is noteworthy that tubular damage begins early in DN [27] and accelerates disease progression and renal function deterioration [28]. Additionally, metabolic disorders, inflammation, and haemodynamic and urinary composition changes associated with diabetes contribute to oxidative stress in renal tubules, leading to the production of various inflammatory cytokines [29]. Thus, in turn, fosters the development and progression of DN. Research has underscored the crucial involvement of dysregulated LncRNAs in DN progression, particularly about the renal tubular injury. Song et al. demonstrated that metastasis-associated lung adenocarcinoma transcript 1 (MALAT1) exacerbates tubular injury in DN by increasing the Lin-28 homolog A levels [27]. SNHG5 attenuates HG-induced inflammation and ROS production by sponging miR-26a-5p, thereby alleviating renal tubular injury [30]. Conversely, NEAT1 aggravates renal tubular injury by regulating mitochondrial autophagy [31].

DANCR, identified as a novel lncRNA, has been previously studied for its potential applications in non-small cell lung cancer [32] and bone differentiation [33]. Given that DN often leads to microvascular diseases, atherosclerosis, and other cardiovascular diseases, DANCR’s involvement in atherosclerosis has been established, and it functions as a predictive biomarker [18]. Additionally, DANCR is associated with unstable angina [28] and plays a role in oxygen-glucose deprivation-induced myocardial injury [19]. Moreover, it regulates vascular smooth muscle cell calcification [34]. DANCR exhibits aberrant expression in acute kidney injury related to sepsis [35] and is associated with immune tolerance in kidney transplantation [23]. Its involvement in the progression of gestational diabetes has also been reported [36]. Notably, a microarray analysis by Guo et al. highlighted differentially expressed LncRNAs between DN tissue and normal kidney tissue, including DANCR [24]. In our current research, higher serum DANCR levels were observed in patients with DN compared to patients with DM and HVs. Moreover, DANCR expression increased in a time-dependent manner in HG-induced HK-2 cells. These findings suggest that dysregulated DANCR may be implicated in DN progression.

To further explore the clinical relevance of dysregulated DANCR in DN, its potential diagnostic capability was initially analysed. Both albuminuria and eGFR were clinical indicators of DN. The correlation between DANCR levels and albuminuria was analysed, and higher DANCR expression was observed in patients with macroalbuminuria (> 300 mg/day) compared to patients with microalbuminuria (30–300 mg/day). Additionally, upon categorizing patients with DN into high eGFR and low eGFR groups based on the mean values, those with low eGFR exhibited higher DANCR levels compared to patients in the high eGFR group. Given these findings, the diagnostic value of DANCR in DN was analysed. Elevated DANCR not only distinguished DM patients with DM from healthy individuals but also differentiated between patients with DM and DN, demonstrating a high diagnostic accuracy. Persistent hyperglycemia contributes leads to renal inflammation, apoptosis, and oxidative stress, closely associated with DN progression. While DANCR has been reported to exhibit aberrant expression in conditions such as novel coronavirus pneumonia infection [22], and osteoarthritis [35], it has also been identified as a regulator of oxidative stress in sarcopenia [37]. In our study, the knockdown of DANCR significantly protected against HG-induced inhibition of renal tubular epithelia cell proliferation increased apoptosis, and the overproduction of inflammation and oxidative stress.

In terms of mechanisms, LncRNAs might function as ceRNAs, to modulate the expression of downstream genes by competitively binding with miRNAs. Therefore, an attempt was made to explore the potential miRNAs and target genes associated with DANCR in DN. Following the confirmation of DANCR presence in the cytoplasm, bioinformatics tools were employed to identify potential binding miRNAs, with particular interest in miR-214-5p. Notably, miR-214-5p exhibited typical inhibition in HG-induced human mesangial cells [38]. The interaction between miR-214-5p and phosphatase and tensin homolog also attenuated glomerular hypertrophy in diabetic conditions [39]. This miRNA was among the 54 dysregulated miRNAs identified in urinary extracellular vesicles from patients with kidney stones [40]. More importantly, screening for dysregulated miRNAs in peripheral blood samples from patients with DN revealed a typical downregulation of miR-214-5p [41]. The targeting relationship between DANCR and miR-214-5p has been demonstrated in atherosclerosis [42]. In our investigation, it was observed that miR-214-5p was typically inhibited in the serum of patients and negative correlation with DANCR levels. The suppression of miR-214-5p counteracted the mitigating effect of low DANCR expression on HG-induced renal tubular epithelial cell injury.

As a transcription factor, KLF5 has been associated with inflammation in mice with renal tubular interstitial injury [43]. Silencing MALAT1 was identified as a regulatory mechanism to alleviate DN pedicle cell injury by modulating KLF5 [44], and KLF5 also serves as a regulator in puromycin-induced apoptosis in renal pedicle cells [45]. Additionally, KLF5 is implicated in lysophosphatidic acid-induced renal tubular fibrosis in DN [46]. Moreover, KLF5 exhibits significant elevation in HG-induced DN renal tubular epithelial HK-2 cells [47]. The targeting relationship between KLF5 and miR-214-5p has been established in non-small cell lung cancer [48]. Consistent with previous studies, KLF5 upregulation was observed in patients with DN and HG-induced HK-2 cells. KLF5 displayed a negative correlation with miR-214-5p and a positive correlation with DANCR.

In summary, DANCR holds diagnostic potential for DN, and the DANCR alleviation of renal tubular injury through the miR-214-5p/KLF5 axis by silencing DANCR presents a novel perspective and approach to managing DN.

### Electronic supplementary material

Below is the link to the electronic supplementary material.

Supplementary Material 1

## Data Availability

No datasets were generated or analysed during the current study.
