# Supplementary figures and images for: Depression of LncRNA DANCR alleviates tubular injury in diabetic nephropathy by regulating KLF5 through sponge miR-214-5p
Source: BMC Nephrol. 2024 Apr 12;25:130. doi: 10.1186/s12882-024-03562-6 (PMC11010359; doi:10.1186/s12882-024-03562-6)

## Supplementary material

KFL5

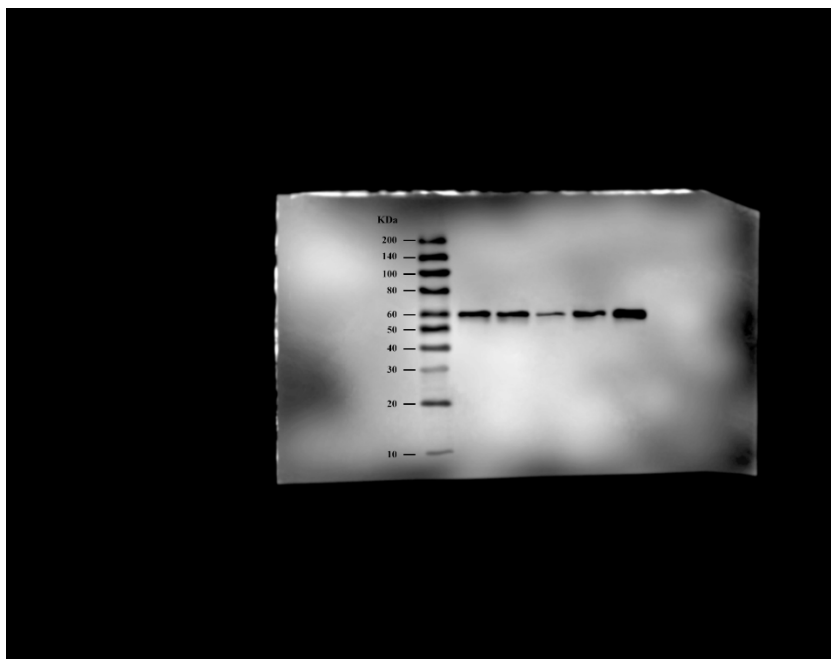

$\beta$ -actin

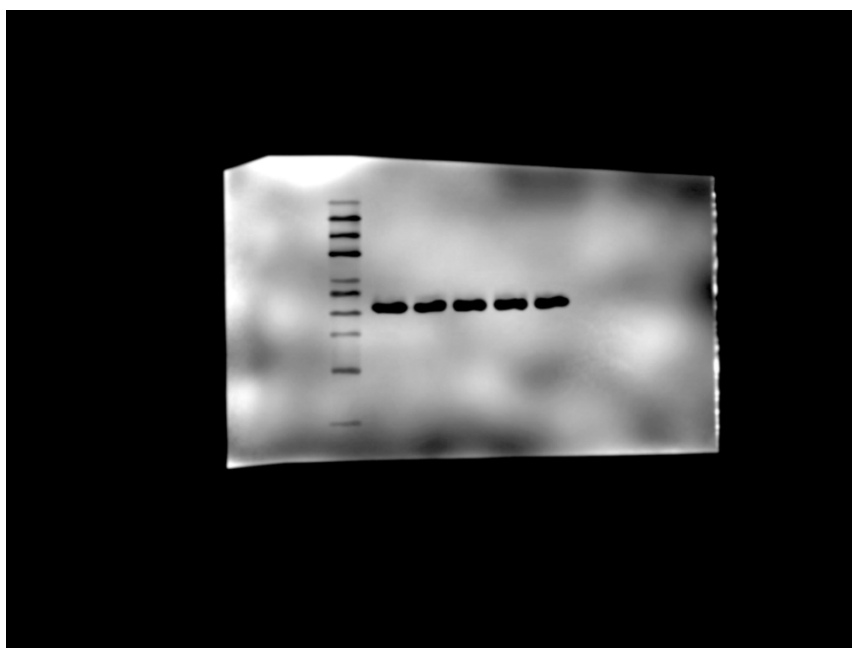

KFL5

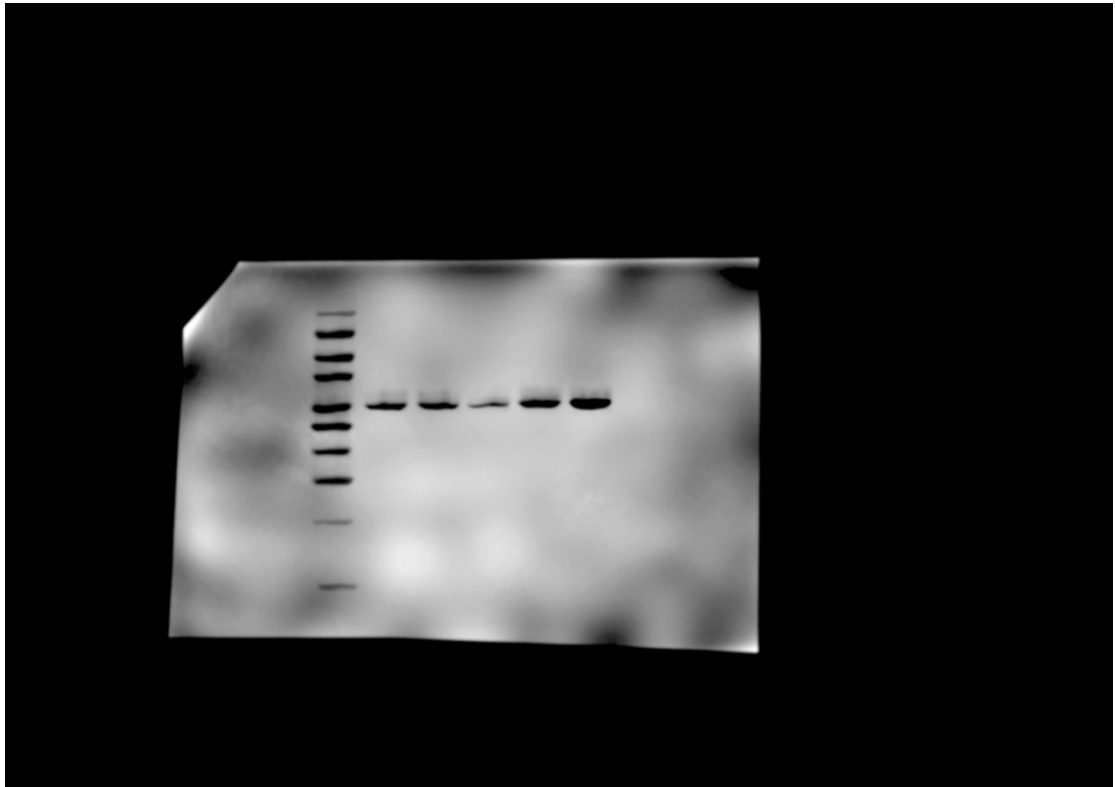

$\beta$ -actin

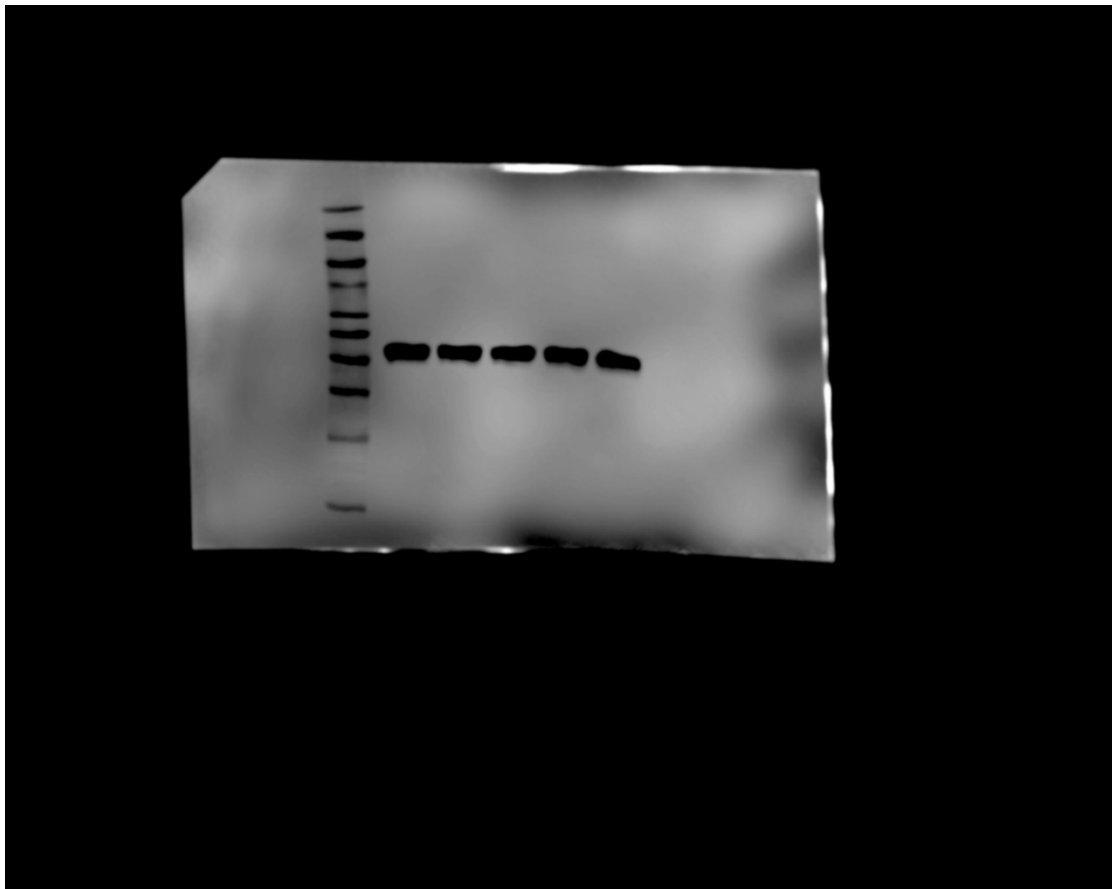

Supplement: Supplementary file 1 — Supplementary Material 1 [file 12882_2024_3562_MOESM1_ESM.pdf]
